# Brachial Plexus Palsy: A Case Report of Compression-Induced Injury Associated With Alcohol Intoxication

**DOI:** 10.7759/cureus.64226

**Published:** 2024-07-10

**Authors:** Hejal Patel, Maleeha Abedi, Afrah Abedi, Twinkle Patel

**Affiliations:** 1 Medicine, Lake Erie College of Osteopathic Medicine, Bradenton, USA; 2 Geriatric Medicine, Loretto Health and Rehabilitation, Syracuse, USA

**Keywords:** chronic alcohol abuse, brachial plexus palsy, brachial plexus injury, alcohol intoxication, brachial plexopathy

## Abstract

Brachial plexus palsy is a complex neuropathy associated with traumatic injuries, inflammatory processes, and tumors. In this report, we present an unusual case of brachial plexus palsy in a 72-year-old man with a history of chronic alcohol abuse. The patient presented to the emergency room with left arm weakness following a fall under the influence of alcohol that resulted in prolonged immobilization. An initial neurologic exam identified paralysis, numbness, and pain in the left upper extremity; however, further studies confirmed the absence of acute fractures or evidence of compartment syndrome. This case highlights the potential for alcohol intoxication and prolonged immobilization, in the absence of traumatic injury, as a contributing cause of brachial plexus palsy. Furthermore, this case emphasizes the importance of considering alternative causes of brachial plexus injury for prompt diagnosis and treatment.

## Introduction

The brachial plexus is a network of nerves originating from the spinal cord that passes through the axilla and extends into the forearm and hand. It is formed from the nerve roots of the cervical nerves C5 to C8 and the first thoracic nerve T1. The nerves formed by the brachial plexus provide sensory and motor innervation to the upper limb, shoulder, arm, forearm, and hand [1].

Causes of injury to the brachial plexus include compression, transection, ischemia, inflammation, metabolic abnormalities, neoplasia, and radiation therapy [2]. Of these, traumatic traction is the most common. Brachial plexus injuries can be further defined as being pre-ganglionic, where the nerve roots are injured at the spinal cord proximal to the dorsal root ganglion, versus post-ganglionic, which is distal to the dorsal root ganglion [3]. Symptoms of brachial plexus injury can vary depending on the severity and location of the injury. Injury can present with symptoms including pain, numbness, and weakness of the upper extremity [2]. 

Brachial plexus palsy in adults is rare; the majority of cases seen are following a motorcycle or automobile accident [4]. Significant trauma to the neck or shoulder region, producing traction on the nerves, is the mechanism of injury seen [1]. The injuries seen in adults are more prevalent in males compared to females between the ages of 15 and 25 [4].

Nerve compression is a relatively uncommon cause of injury since there is a significant bony structure in this region, including the vertebrae, clavicle, scapula, and first rib [5]. In unusual cases, brachial plexus palsy has presented as a result of alcohol intoxication [6,7]. We present a case of a 72-year-old man with a long-standing history of chronic alcohol abuse who sustained brachial plexus palsy following a fall, during which he became entrapped behind a toilet for 12 hours. Subsequent prolonged compression and swelling of the upper extremity caused him to develop palsy of his left upper extremity. This case report highlights the importance of considering alternative causes of brachial plexus injury for prompt diagnosis and treatment.

## Case presentation

A 72-year-old male with an ongoing history of chronic alcohol abuse was brought into the Emergency Department by emergency medical services. He was found by his daughter in his bathroom, wedged behind the toilet and the wall following a fall. It was estimated that he had been passed out, stuck behind the toilet with his left arm pinned in an abducted position for 12 hours. The toilet was removed to free the patient and bring him to the emergency room. He presented to the emergency room with left arm paralysis, left arm numbness, shoulder pain, chest pain, and coffee-ground emesis. He self-reported that he drinks around three drinks of rum a day; however, his daughter believed that it was more than this. 

On evaluation in the emergency room, he had severe edema and bruising of the left upper extremity. He was found to have bluish discoloration of the left arm with increased pain with a passive range of motion, but pulses were intact.

At admission, the patient's vital signs were as follows: temperature 36.8°C, pulse 111 bpm, blood pressure 135/93 mmHg, respirations 20 per minute, and pulse oximetry of 95%. The following labs were obtained for the patient: metabolic panel, creatine kinase (CK) isoenzymes, complete blood count (CBC) with differential, liver function tests, and ethanol level. Pertinent labs upon the patient's presentation can be seen in Table 1. 

**Table 1:** Laboratory values All other values are within normal limits BUN: Blood urea nitrogen; CK: Creatine kinase

Parameter	Laboratory value	Reference range
Direct bilirubin	0.6 mg/dL	0.0-0.3 mg/dL
Total bilirubin	1.6 mg/dL	0.0-1.0 mg/dL
Aspartate aminotransferase	51 U/L	13-40 U/L
Alanine transaminase	63 U/L	9-40 U/L
Ethanol level	15 mg/dL	0-9 mg/dL
Creatinine	1.58 mg/dL	0.70-1.30 mg/dL
CK	323 U/L	46-171 U/L
BUN	25 g/dL	9-23 g/dL
White blood cell count	11.3 10^3/µL	4.1-11.0 10^3/µL
Mean corpuscular volume	98.3 fL	80.0-95.0 fL
Mean corpuscular hemoglobin	32.7 pg	27.0-32.0 pg
Mean corpuscular hemoglobin concentration	33.2 g/dL	32.0-36.0 g/dL
Red cell distribution width	13.3%	10.5-14.5%
Platelet count	580 10^3/µL	150-450 10^3/µL

**Figure 1:**
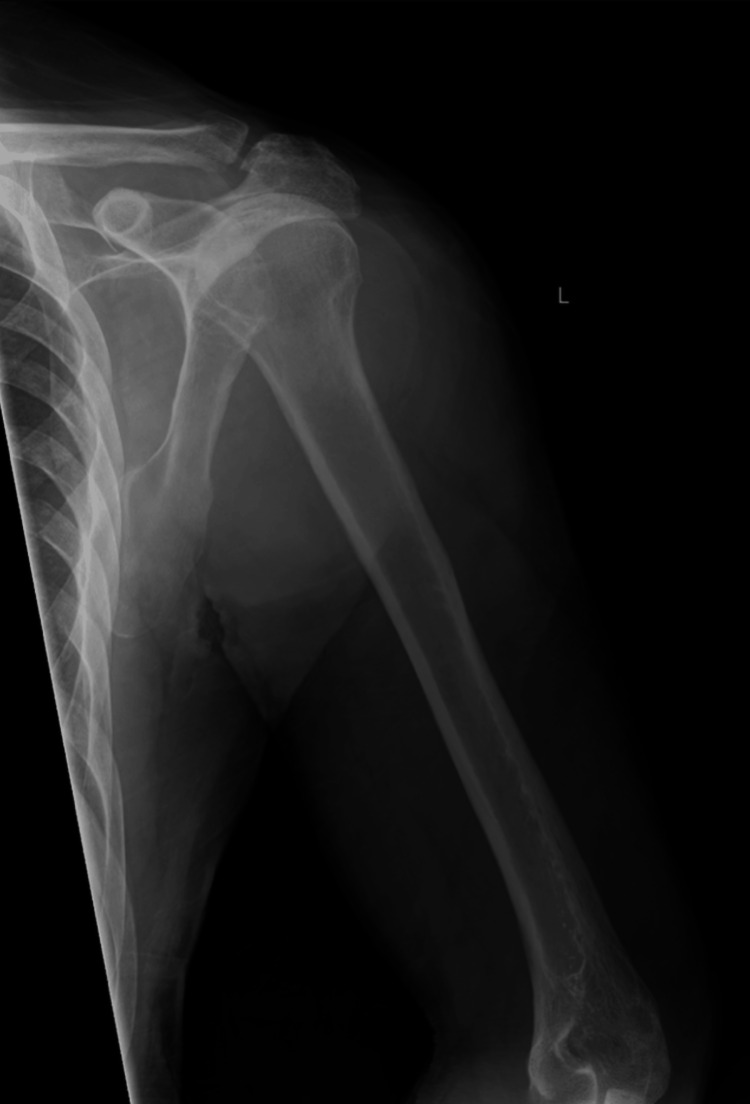
X-ray of the left shoulder and left upper arm Negative for any fractures or shoulder dislocation

**Figure 2:**
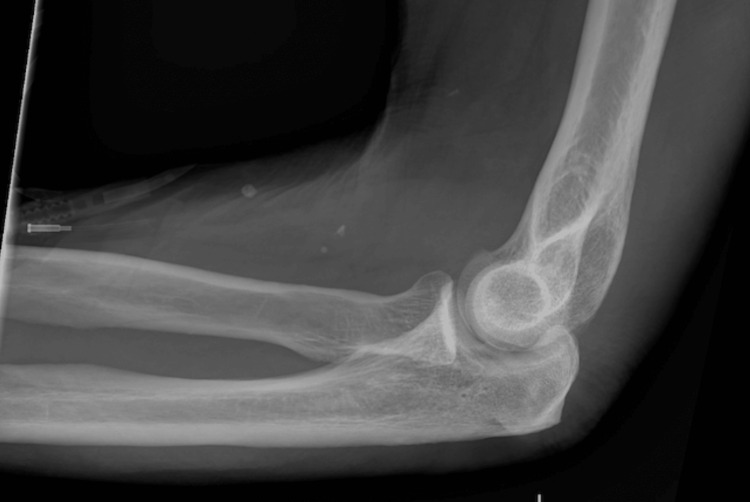
X-ray of the left forearm Negative for any fractures

**Figure 3:**
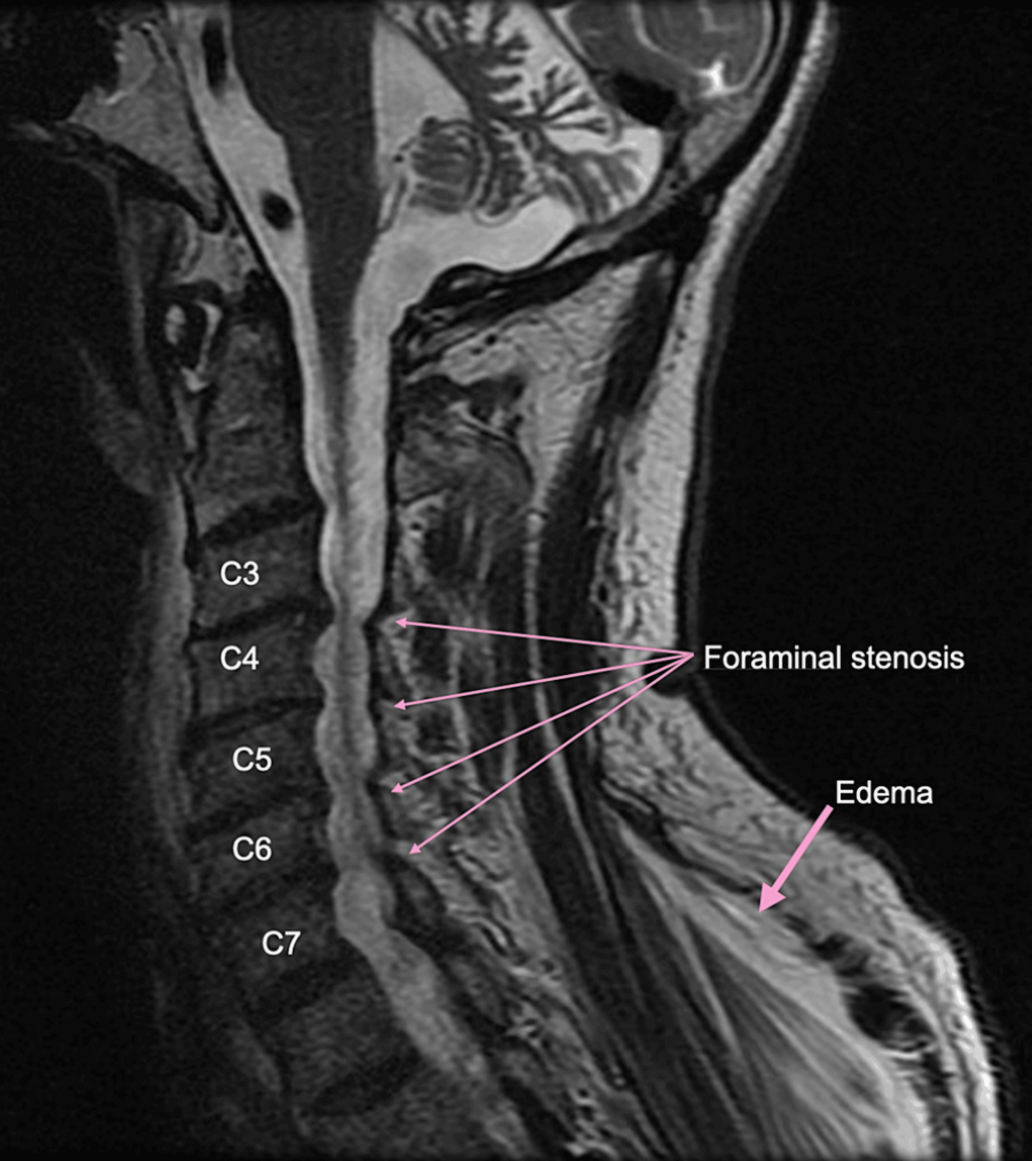
MRI cervical spine without contrast Spondylosis has resulted in multilevel mild spinal canal stenosis and moderate to severe neural foraminal stenosis from C3 to C7. There is no evidence of cord compression. Additionally, there is evidence of edema in the back and shoulder musculature, along with cerebellar volume loss.

**Figure 4:**
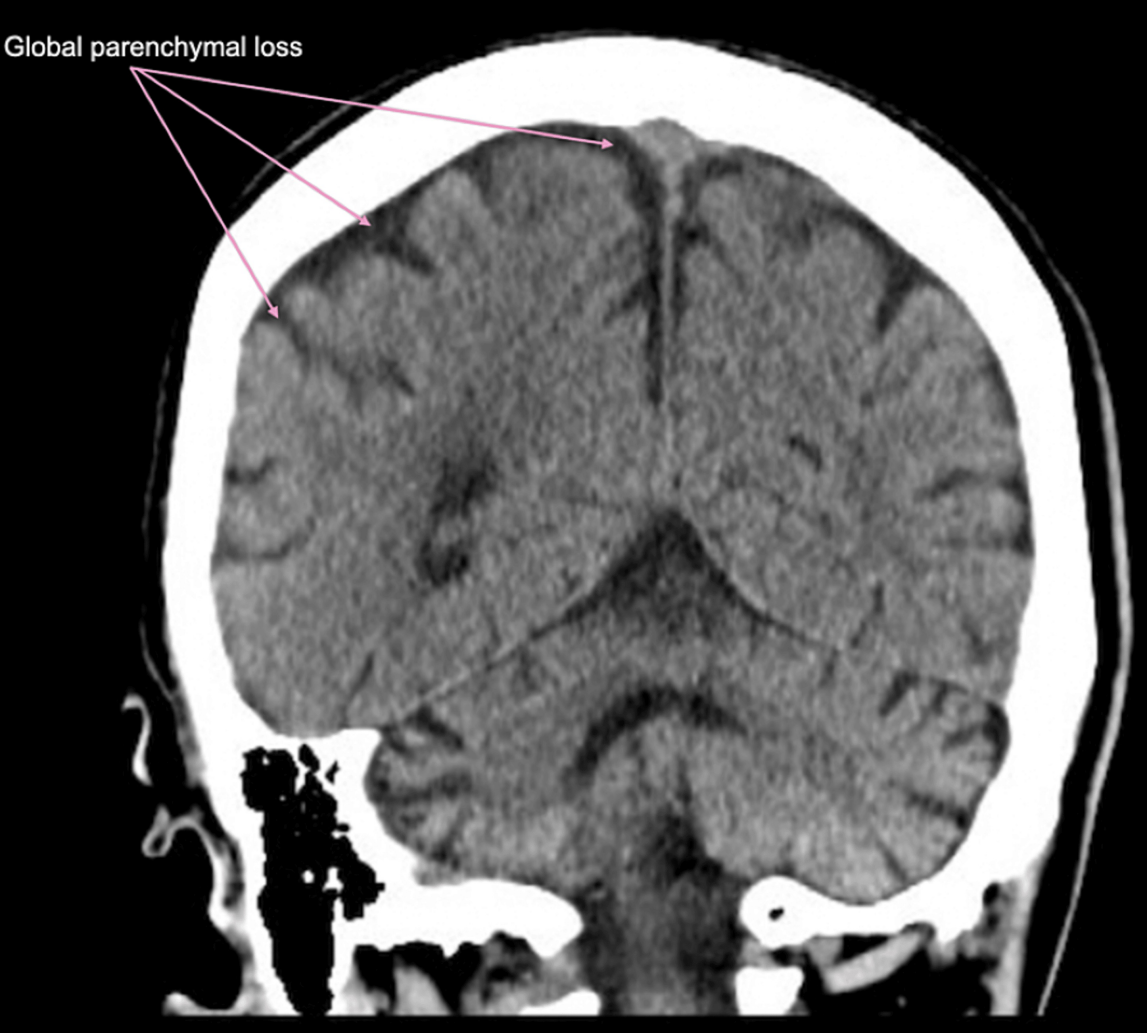
CT head without contrast There was no evidence of acute territorial infarction, parenchymal hemorrhage, or mass. There was mild-moderate global parenchymal volume loss.

He was admitted with a diagnosis of severe rhabdomyolysis, acute tubular necrosis, and left brachial plexus palsy. At the time of presentation, he was hemodynamically stable, and any acute fractures and compartment syndrome were ruled out. Initial X-rays of the left shoulder, upper arm, and forearm were negative for any acute findings (Figures 1-2). Magnetic resonance imaging (MRI) of the cervical spine displayed evidence of chronic degenerative changes but was negative for any acute findings (Figure 3). There was evidence of cerebellar volume loss, a chronic change consistent with alcohol abuse. Additionally, an initial CT of the brain ruled out any acute findings of a stroke (Figure 4). In the absence of acute findings on imaging, it was deduced that his injuries were likely related to prolonged compression and subsequent edema.

He was medically stabilized and discharged to a short-term rehabilitation facility. The patient was evaluated at the facility to have complete paralysis of the left upper arm with no ability to move the shoulder, elbow, wrist, or fingers. During his rehabilitation course, he developed worsening contractures of the distal extremity. He lacked any active range of motion of the left extremity; his left shoulder and elbow could be passively moved on physical exam. However, his wrist, metacarpophalangeal, and proximal interphalangeal joints remained flexed with no active or passive range of motion. The patient had a loss of all sensation to the affected extremity.

## Discussion

This case emphasizes the significance of considering alternative causes of brachial plexus palsy, especially in cases involving alcohol intoxication. While initially, the patient's presentation may lead the clinician to suspect compartment syndrome, nerve injury due to fracture, or stroke, it is essential to consider alternative causes of brachial plexus palsy for prompt diagnosis [8-10]. Clinicians should suspect brachial plexus palsy in cases of prolonged sustained compression injury, even in the absence of traumatic injury-producing traction.

Many cases of alcohol intoxication leading to "Saturday Night Palsy" have been reported. "Saturday Night Palsy" is a colloquial term referring to what is known as compressive radial mononeuropathy [11,12]. Compressive radial mononeuropathy is a brachial plexopathy resulting from temporary compression and stretching of the arm against an object following alcohol or drug use [12]. It results from sleeping with the arm in an unusual position that compresses the radial nerve roots [11,12]. The patient will present with numbness, weakness, and tingling in the dorsal forearm and hand. On exam, there will be a loss of extensor muscle function, resulting in a wrist drop; however, the function of the flexor muscles of the hand remains intact [12]. Such cases with more localized neurologic deficits can improve with conservative measures such as physical therapy and splinting. As described, unlike the case we have presented here, this is mononeuropathy that does not involve a large portion of the brachial plexus. 

The injury in this patient resulted from compression of the brachial plexus nerve roots, specifically prolonged rotation of the head in the setting of immobilization and swelling of the upper extremity. The patient clinically presented with extensive loss of function in the left upper extremity, suggesting that multiple nerve roots were involved [1]. However, the patient refused electromyography (EMG) studies while admitted; as a result, it is a limitation in our ability to specifically identify which nerve roots or portion of the brachial plexus was involved. Based on the patient's exam findings of complete loss of motor and sensory function in the extremity and in the absence of acute injury to the cervical nerve roots on imaging, we can speculate that the patient had extensive damage at the level of the brachial plexus cords [1,2,4]. 

Additionally, prolonged immobilization can result in rhabdomyolysis, as seen in this patient [13]. While rhabdomyolysis may not directly cause a brachial plexus injury, this condition can compromise blood flow to the affected area, causing localized ischemia and further exacerbating paralysis [7]. The patient also had significant upper extremity edema that further compromised adequate circulation to the affected extremity. 

The effects of chronic alcohol use can result in peripheral symmetric neuropathy in a stocking-and-glove distribution, in part due to subsequent nutritional deficiencies and direct neurotoxic effects of alcohol [14]. In this case, the patient's findings were more consistent with an acute injury of the brachial plexus rather than the chronic effects of alcohol use.

​​Silber et al. report two cases of brachial plexopathy related to compression after alcohol intoxication [6]. The first patient they reported presented with rhabdomyolysis following prolonged immobilization. However, the patient's left arm was described to be almost fully functional following rehabilitation. The second patient was a known alcoholic who went to bed with the arm in an unusual position; she similarly presented with a brachial plexopathy that improved with rehabilitation in four weeks. In this case, we report severe brachial plexopathy that was not responsive to intensive physical therapy and rehabilitation and progressively worsened despite these measures. 

Anwar et al. report a case of bilateral brachial plexus palsy following alcohol intoxication and prolonged immobilization [7]. The patient similarly had poor functional outcomes despite four months of rehabilitation. Unlike the case presented here, the patient also presented with an alcohol-related brain injury. However, they concluded that it was likely compression-related in the absence of acute findings on head CT supporting the palsy. 

While this is an unusual case, the incidence of brachial plexus palsies in adults may be higher; however, they remain poorly reported due to unusual attributing causes. It is necessary to recognize that prolonged compression could also contribute to such injuries. 

## Conclusions

Current medical understanding suggests that trauma is the main cause of brachial plexus palsy. However, compression-related injuries may represent a more significant portion of these palsies. This case report highlights the associations between chronic alcohol consumption and the risk of nerve injury. The prolonged compression of the brachial plexus following a fall, compounded by alcohol-induced sedation and subsequent rhabdomyolysis, demonstrates the multifactorial nature of brachial plexus injuries. Medical professionals should maintain a high index of suspicion for such injuries, even in the absence of traumatic injury. Identifying unique causes of brachial plexus palsies can help to assess and treat affected patients accurately.
